# Supplementary material for: The Angiogenesis Inhibitor Isthmin-1 (ISM1) Is Overexpressed in Experimental Models of Glomerulopathy and Impairs the Viability of Podocytes
Source: Int J Mol Sci. 2023 Feb 1;24(3):2723. doi: 10.3390/ijms24032723 (PMC9916724; doi:10.3390/ijms24032723)
Supplement: Supplementary file 1 [file ijms-24-02723-s001.zip › ijms-2097811-supplementary.pdf]

SUPPLEMENTARY DATA

TABLE S1

Gene name	Direction Primer sequence (5′-3′)
**β5 (rat and mouse)**	*fwd:* ACCTGCCAAGATGGCATATC<br>*rev:* CACGGACACTTCAAAGGATG
**GRP78 (rat)**	*fwd:* CCGTAACAATCAAGGTCTACGA<br>*rev:* AAGGTGACTTCAATCTGGGGTA
**GRP78 (mouse)**	*fwd:* CTGAGGCGTATTTGGGAAAG<br>*rev:* TCATGACATTCAGTCCAGCAA
**ISM1 (rat and mouse)**	*fwd:* TCCAGATCTTTCCAAAGCTGAT<br>*rev:* GCCATCAACCACCTCTATGG
**IL6 (mouse)**	*fwd:* CCAGGTAGCTATGGTACTCCAGAA<br>*rev:* TGCCTTCATTTATCCCTTGAA
**HPRT (mouse)**	*fwd:* GGAGCGGTAGCACCTCCT<br>*rev:* CTGGTTCATCATCGCTAATCAC
**HPRT (rat)**	*fwd:* GACCGGTTCTGTCATGTCG<br>*rev:* ACCTGGTTCATCATCACTAATCAC
**Gusb (Mouse)**	*fwd:* CTCTGGTGGCCTTACCTGAT<br>*rev:* CAGTTGTTGTCACCTTCACCTC

Table S1. Gene-specific primer sequences used for RT-qPCR

TABLE S2

Up-regulated genes				Down-regulated genes		
**Symbol**	**Fold Change**	**p-value**		**Symbol**	**Fold Change**	**p-value**
HAVCR1	36,082	7.42E-08		Slc7a12	-9,996	1.35E-03
SERPINA3	23,150	1.28E-03		RGD1563294	-6,425	3.16E-05
FGB	18,605	1.92E-07		Akr1c12	-5,578	1.76E-03
GPNMB	8,954	7.32E-06		RGN	-5,501	7.61E-04
HMOX1	8,562	2.21E-03		Slco1a1	-5,164	1.06E-02
IGFBP1	7,673	1.06E-05		CACNG5	-5,028	5.68E-03
ALOX15	7,133	1.35E-03		HNMT	-5,010	5.81E-04
PDK4	6,936	1.05E-06		SLC7A13	-4,980	1.50E-03
ADAMTS1	6,929	1.53E-06		PRIMA1	-4,795	4.32E-03
GPX2	6,715	1.43E-03		HRG	-4,737	1.98E-03
ANGPTL4	6,471	4.85E-07		Olfr136	-4,648	1.83E-02
S100A8	6,372	2.49E-02		GC	-4,339	6.88E-03
SPP1	6,313	1.48E-06		Cml1	-4,216	7.29E-04
HMGCS2	6,023	3.00E-03		Ces1e	-4,207	5.15E-04
SERPINE1	5,975	6.62E-06		Slco1a6	-3,863	5.63E-03
FGA	5,805	4.59E-05		Olr1408	-3,808	1.29E-02
TIMP1	5,453	3.26E-06		Kap	-3,754	3.25E-02
CLU	5,208	7.98E-06		CYP2C9	-3,724	2.40E-02
SLC34A2	5,155	1.61E-05		GUCY1B2	-3,631	7.38E-05
FGG	5,067	1.06E-05		CHTF18	-3,572	4.34E-03
LAMC2	4,874	4.02E-05		SPATA22	-3,511	1.25E-04
FAM129A	4,755	1.03E-08		SLCO4C1	-3,498	2.77E-04
CDKN1A	4,742	6.29E-05		AFM	-3,472	1.59E-03
RASD1	4,539	3.07E-05		MLC1	-3,426	3.18E-03
BTG2	4,533	2.45E-03		SLC22A25	-3,407	1.28E-03

Table S2: Top 25 up- and down-regulated genes in L-NAME model between W0 and CTL groups.

TABLE S3

	UAER			UPER			Albuminemia		
	**CTL**	**model**	**p**	**CTL**	**model**	**p**	**CTL**	**model**	**p**
PAN	0.00±0.000	2.42±0.15	<0.001	0.06±0.004	12.71±0.75	<0.001	14.9±0.65	4.38±0.35	<0.001
DOXO	0.00±0.000	3.02±0.12	<0.001	0.15±0.01	9.18±0.51	<0.001	16.47±0.57	4.25±0,16	<0.001
ob/ob	0.002±0.001	0,056±0.008	<0.001						
LPS	0.04±0.01	0.27±0.04	<0.001						

Table S3. Biochemical parameters in animal models of glomerulopathy. UAER (g/mmol), UPER (g/mmol) and albuminemia (g/L) in PAN and DOX models. UAER (g/mmol) in ob/ob model and UAER (g/g) in LPS model. . UAER: Urinary albumin excretion ratio and UPER: Urinary protein excretion ratio.

TABLE S4

(*DOWNLOAD EXCEL FILE*)

Table S4: Genes related to ISM1 regulation during renal regeneration. This table describes the statistics identified by the supervised Pavlidis template matching algorithm on ISM1 expression during renal regeneration. For each column of significant genes: correlation coefficient rvalues, raw p-values and False Discovery Rate adjust qvalues.

FIGURE S1

A)

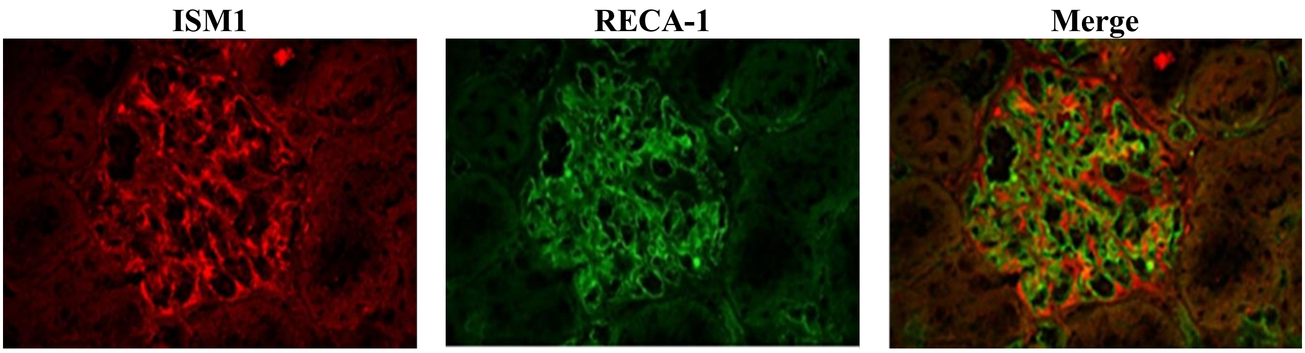

B)

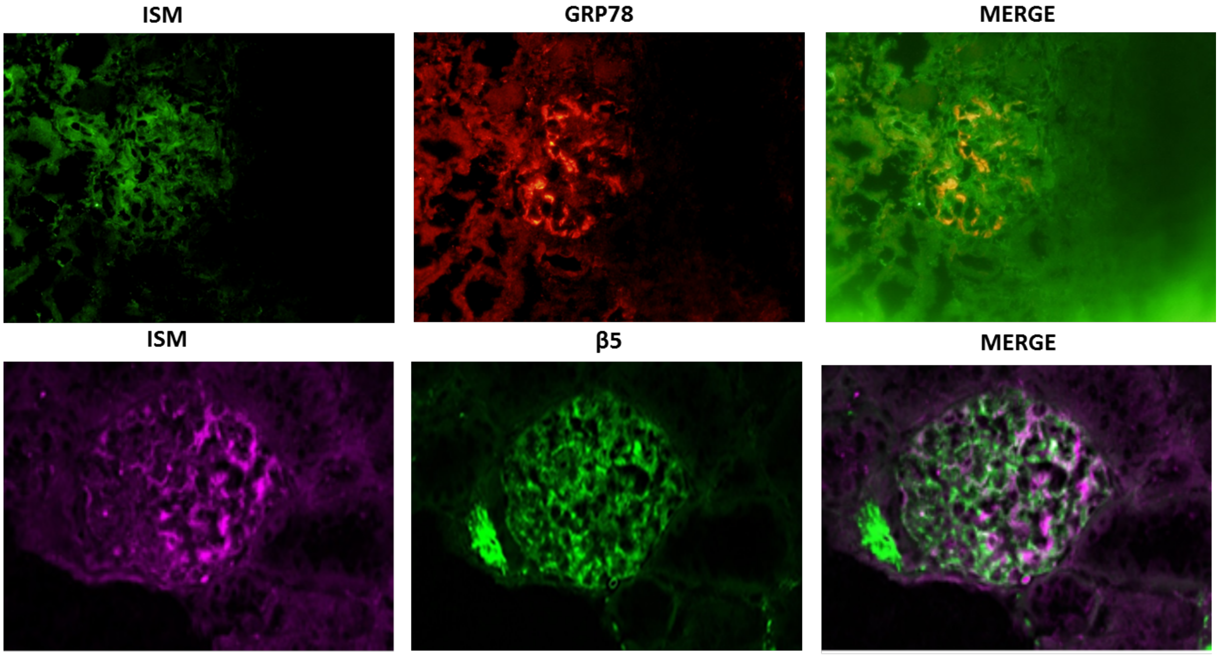

Figure S1. Immunohistochemical analysis of co-localization of ISM1: A) ISM1 does not colocalize with RECA-1, a rodent pan-endothelial cell marker. B) Co-localization of ISM1with its receptors, GRP78 and beta5 integrin in rats.
